# No-Show Rates at a Plastic Surgery Clinic: Insights From Appalachian Healthcare Systems

**DOI:** 10.7759/cureus.76873

**Published:** 2025-01-03

**Authors:** Armein Rahimpour, Emily Saurborn, Abigail Murphy, Jentre H Hyde, Andrew Weaver, Semeret Munie, Paul Bown, Rahman Barry

**Affiliations:** 1 General Surgery, Marshall University Joan C. Edwards School of Medicine, Huntington, USA; 2 Trauma and Surgical Critical Care, Marshall University Joan C. Edwards School of Medicine, Huntington, USA; 3 Bariatric Surgery, Marshall University Joan C. Edwards School of Medicine, Huntington, USA; 4 Plastic and Reconstructive Surgery, Marshall University Joan C. Edwards School of Medicine, Huntington, USA

**Keywords:** appalachia, appointment adherence, no-show rates, patient engagement, plastic surgery clinics, predictors of attendance, resource optimization, rural healthcare disparities, socioeconomic barriers

## Abstract

Introduction: Missed appointments, or "no-shows," occur when patients fail to notify the healthcare clinic of their inability to attend. No-show appointments place a significant burden on healthcare systems, costing clinics hundreds of thousands of dollars annually. Not only do patients miss out on receiving care, but prospective patients also face longer wait times due to appointment vacancies. This study aims to evaluate the factors associated with patient no-shows at a rural plastic surgery clinic.

Methods: Two-sample t-tests were used to compare age, BMI, weather temperature, and distance from the clinic between show and no-show groups. Chi-square tests of independence assessed the relationship between categorical variables- such as gender, clinic time (AM vs. PM), weather conditions, appointment type, insurance type, smoking status, and race- and appointment attendance. The Mann-Whitney U test analyzed the distribution of post-operative visit numbers between the show and no-show groups. To control for potential confounders, a multivariate logistic regression model was used to assess the independent effects of demographic, environmental, and procedural factors on no-show rates.

Results: The mean age of patients in the no-show group was 40.73 years (SD = 14.08), compared to 42.85 years (SD = 12.48) for the show group. Higher no-show rates were significantly associated with male gender (p<0.01), impending weather (p<0.001), appointment type (new patient, pre-operative, post-operative) (p<0.001), and self-pay appointments (p<0.01). In addition, facial fracture follow-ups and facelift/blepharoplasty procedures showed significance (p<0.001). After adjusting for cofounders, male patients had significantly higher odds of missing their appointments compared to female patients (OR = 1.85, 95% CI: 1.12-3.06, p = 0.014). Post-operative patients were also more likely to miss appointments than new patients (OR = 1.92, 95% CI: 1.25-2.95, p = 0.003), while pre-operative patients showed an increased likelihood of no-show, though this did not reach statistical significance (OR = 1.45, p = 0.08). Weather conditions remained a significant factor after adjusting for other variables. Cloudy weather was associated with higher no-show rates compared to fair weather (OR = 1.67, 95% CI: 1.20-2.34, p = 0.002), whereas light rain did not significantly affect attendance (p = 0.38). Additionally, facial procedures were linked to significantly lower no-show rates compared to body contouring procedures (OR = 0.48, 95% CI: 0.29-0.79, p = 0.004).

Discussion and conclusion: No-show rates are influenced by demographic and environmental factors. Male gender, insurance type, and weather conditions were significantly associated with higher no-show rates, while age, BMI, and distance from the clinic had mixed or non-significant associations. Future studies should explore the role of telemedicine in improving patient attendance in plastic surgery clinics.

## Introduction

Missed appointments, or "no-shows," are those that patients neither keep nor cancel [1]. Patients who no-show their appointments fail to notify the healthcare clinic of their miss. This failure to notify can lead to significant healthcare costs and frustration for both providers and other patients [2]. Healthcare centers can lose hundreds of thousands of dollars each year due to patient no-shows [3]. A no-show rate of 67,000 appointments per year has been shown to cost healthcare centers nearly $7 million [4]. This significant cost places a large burden on healthcare centers, ultimately affecting patient care. Wait-listed patients may report dissatisfaction with long wait times and opt to attend another practice, further contributing to the healthcare costs of no-shows [2]. Addressing no-show rates is a critical step toward maximizing healthcare delivery in underserved areas.

No-show appointments present a critical challenge to healthcare delivery in all settings, and different geographic areas present their own unique challenges. Appalachia is a rural region in the eastern United States extending from Pennsylvania to Alabama. With its predominantly rural geography, high poverty rates, and limited healthcare infrastructure, the region faces unique barriers to equitable care delivery [5]. The high poverty rates in this area limit access to private insurance and affordable healthcare for residents [6]. Literature suggests that patients with lower income are more likely to miss their appointments [7]. Many patients face additional challenges, such as the inability to take time off work, lack of insurance, and low health literacy, all of which contribute to higher rates of no-shows [8]. Limited access to rural clinics, combined with a high patient-to-doctor ratio and limited insurance coverage, exacerbates the issue of patient no-shows. It is imperative for clinics in the region to address these contributing factors [5,6].

Transportation barriers are a significant factor contributing to no-show rates in rural Appalachia [9]. The mountainous terrain with winding roads significantly delays travel times, resulting in healthcare appointments consuming a whole day for those travelling from a rural location [5]. Public transportation is limited in Appalachia, and oftentimes, patients are required to have a private vehicle in order to travel [5]. Studies show that patients who travel longer distances are significantly more likely to miss their appointments [7]. Assisting patients in finding alternative transportation and ensuring service during adverse conditions has been shown to improve comprehensive healthcare delivery [10].

Environmental factors such as adverse weather conditions play a role in a patient’s ability to attend their scheduled appointment. Conditions such as snow and heavy rain further complicate travel for patients. In Appalachia, the region’s rural infrastructure and mountainous terrain make it particularly susceptible to mudslides, snow closures, and flooding, further impacting appointment adherence [5]. For clinics in Appalachia, understanding the role of weather conditions on travel times is essential to reducing no-show rates and ensuring equitable access to care.

To reduce the burden of patient no-shows, clinics can implement interventions such as targeted appointment reminders, flexible scheduling, transportation assistance, and telemedicine options [11]. These strategies have been shown to reduce no-shows in similar resource-limited settings and improve clinical efficiency. Additionally, predictive modeling and data analytics can help identify patients at higher risk of missing appointments, allowing clinics to tailor notifications and scheduling for these individuals [12].

The purpose of this study is to investigate the factors contributing to patient no-show appointments in Appalachia, a region with unique barriers to healthcare access. This study was conducted at an academic plastic surgery clinic affiliated with the Joan C. Edwards School of Medicine Department of General Surgery at Cabell Huntington Hospital, in Huntington, West Virginia. The clinic specializes in a variety of plastic and reconstructive surgeries ranging from both aesthetic procedures to oncoplastic reconstruction. This study provides an analysis of the unique setting within the Appalachian region, where patients have limited access to specialized care. 

The study aims to provide insights for improving clinic efficiency and resource management, which may be implemented by other rural providers and healthcare systems. By addressing the factors contributing to no-show appointments, healthcare providers in Appalachia can better allocate their time and resources, ensuring optimal patient care. This is particularly critical in regions with limited healthcare access, where missed appointments can delay treatment for both current and waitlisted patients.

## Materials and methods

The study received approval from the Marshall University Institutional Review Board (2255170-1). Patient records were retrospectively reviewed from our registry at the plastic surgery office affiliated with Marshall University, the Joan C. Edwards School of Medicine. This is an academic teaching hospital, regional referral center, and American College of Surgeons verified Level-2 Trauma Centers in Huntington, West Virginia.

This study included a retrospective analysis of patient appointment data from a plastic surgery clinic located in rural Appalachia. Data collection occurred between October 2022 and October 2024. Data were extracted from the clinic's electronic medical records and organized into two datasets: attendance and census data, and demographics and appointment details. Attendance and census data included daily appointment metrics consisting of patients who attended their appointments, missed their appointments and the total number of appointments scheduled. No-show rate was calculated as a ratio of missed appointments to the total scheduled appointments for the day. Demographics and appointment details were also collected and included age, gender, ethnicity, smoking status, BMI, and zip code. Distance from the clinic was calculated using Google Maps, measuring the shortest driving route from the patient’s zip code and clinic. Documented appointment details included time of day (AM: 8-12, PM 1-5); weather conditions sourced from historical data using Weather Underground, appointment type (new, pre-operative, post-operative), and reason for appointment. Insurance type was categorized as self-pay or other types of coverage. For post-operative visits, details of the surgical procedure and number of follow-ups were noted. The control group consisted of one patient who attended their appointment and was randomly selected from both the morning and afternoon sessions for each clinic day.

Two-sample T-tests were used to compare age, BMI, weather temperature, and distance from the clinic between show and no-show groups. Chi-square tests of Independence assessed the relationship between categorical variables such as gender, clinic time (AM vs PM), weather conditions, appointment type, insurance type, smoking status, and race with appointment attendance. Mann-Whitney U Test analyzed the distribution of post-operative visit numbers between show and no-show groups. Furthermore, a multivariate logistic regression analysis was performed for statically significant variables, with patient attendance (show vs. no-show) as the dependent variable. Statistical significance was calculated at a 95% confidence interval, with a p-value threshold of <0.05. The study was IRB-approved and all patients provided informed consent.

## Results

Our analysis of one plastic surgeon in the Appalachia region revealed a total of 2,002 appointments. Furthermore 1,725 patients completed their visits ("shows") and 277 missed their appointments ("no-shows"). The overall no-show rate for our plastic surgery clinic was 13.84%. On average for each clinic day the plastic surgeon saw 18.5 patients total and three patients failed to show up, for total census of 21.5 for each clinic day. 

Table 1 shows overall descriptive statistics for various factors comparing individuals from the show and no-show groups. As seen in Table 1 the mean age of patients who attended and did not attend their appointments were 42.85 years and 40.73, respectively. The mean temperature (measured in Fahrenheit) for patients who attended and did not attend their appointments were 65.03 and 64.22, respectively. The mean BMI for those who attended was 28.85, compared to 28.71 for no-shows. Finally, patients who attended appointments traveled an average distance of 34.49 miles, compared to 39.98 miles for those who did not attend. 

**Table 1:** Descriptive Statistics Based on Appointment Attendance. StdDev; Standard Deviation. Conf; Confidence Interval 95%. Following is the measure for each variable: Age (years), Weather (Fahrenheit), and Distance from clinic (miles).

Descriptive Statistics	Age (Show)	Age (No-show)	Weather (Show)	Weather (No-show)	BMI (Show)	BMI (No-show)	Distance from clinic (Show)	Distance from clinic (No-show)
Mean	42.85	40.73	65.03	64.22	28.85	28.71	34.49	39.98
Median	42	39	67	65.5	28	28	28.5	25
Mode	34	30	78	78	29	27	7	6
StdDev	12.48	14.08	16.1	16.72	6.61	6.65	31.85	50.92
Range	59	80	74	74	43	41	163	575
Min	19	8	24	24	17	15	0	0
Max	78	88	98	98	60	56	163	575
Count	181	276	181	276	179	209	180	264
Conf	1.83	1.67	2.36	1.98	0.97	0.91	4.68	6.17

Table 2 shows the chi-square analysis. This was conducted to examine associations between patient attendance (show vs. no-show) and various categorical variables.

**Table 2:** Chi Square Analysis Between Show and No-Show Groups. Data presented as n (%), where n is number of patients and % percentage. P-value threshold of < 0.05 was for statistical significance.

Chi square	Show	No-Show	p-value	χ²
Gender			0.0078	7.12
Male	22 (12)	62 (22)		
Female	159 (88)	214 (88)		
Clinic Time			0.602	0.263
AM	93 (51)	134 (48)		
PM	88 (49)	142 (52)		
Patient Type			<0.001	20.43
Pre-operative	19 (9)	41 (15)		
Post-operative	38 (17)	92 (33)		
New	162 (74)	142 (52)		
Cigarette Use			0.062	3.474
Yes	15 (8)	33 (15)		
No	163 (92)	186 (85)		
Weather (F)			p < 0.001	0.78
Fair	104 (58)	155 (56)		
Other	6 (3)	5 (2)		
cloudy	58 (32)	100 (36)		
light rain	12 (6)	16 (6)		
Insurance Type			p < 0.001	19.07
Self pay	94 (52)	67 (30)		
Other	87 (48)	156 (70)		
Race			0.116	2.47
White	175 (97)	227 (93)		
Other	6 (3)	18 (7)		

A significant association was observed between gender and attendance (χ² = 7.12, p=0.0078). Males accounted for 22 (12%) shows and 62 (22%) no-shows, while females comprised 159 (88%) shows and 214 (88%) no-shows.

Morning appointments had 93 (51%) shows and 134 (48%) no-shows, while afternoon appointments had 88 (49%) shows and 142 (52%) no-shows. The chi-square statistic was 0.263, with a p-value of 0.602, indicating that appointment timing was not a determining factor in no-shows.

The type of appointment significantly influenced no-show rates (χ² = 20.43, p < 0.001). Pre-operative patients had 19 (9%) shows and 41 (15%) no-shows, post-operative patients had 38 (17%) shows and 92 (33%) no-shows, and new patients accounted for 162 (74%) shows and 142 (52%) no-shows. New patients were more likely to attend, whereas pre-operative and post-operative appointments showed higher no-show rates.

Post-operative patients with multiple follow-up visits were particularly prone to missing appointments. Furthermore for postoperative visit numbers and appointment attendance a Mann-Whitney U test was conducted. This group included only portion of patients who were postoperative. The median postoperative visit number for the No-Show group was 2, compared to 1 for the Show group. The U statistic was 2325, with a p-value of 0.0037, indicating a significant difference between the two groups. Patients with more postoperative visits were more likely to miss their appointments.

Although smokers exhibited a trend toward higher no-show rates, the difference did not reach statistical significance (χ² = 3.474, p = 0.062). Among smokers, there were 15 (8%) shows and 33 (15%) no-shows, while non-smokers had 163 (92%) shows and 186 (85%) no-shows.

A significant association was found between weather conditions and attendance (p<0.001). Fair weather corresponded to 104 (58%) shows and 155 (56%) no-shows, cloudy weather to 58 (32%) shows and 100 (36%) no-shows, and light rain to 12 (6%) shows and 16 (6%) no-shows. Cloudy weather was most strongly associated with no-shows, while fair weather had higher show rates.

A significant association was observed between insurance type and appointment attendance (p < 0.001). Patients with self-pay insurance were more likely to attend their appointments compared to those with other types of insurance. Self-pay patients had 94 (52%) shows and 67 (30%) no-shows, while patients with other insurance types had 87 (48%) shows and 156 (70%) no-shows.

No significant association was found between race/ethnicity and appointment attendance (χ² = 2.47, p = 0.116). However, individuals categorized as "Other" showed a trend toward higher no-show rates. White patients comprised 175 (97%) shows and 227 (93%) no-shows, while patients of other races accounted for six (3%) shows and 18 (7%) no-shows.

Table 3 shows the analysis for the t-test. A two-sample t-test was conducted to evaluate differences between patients who showed up for their appointments and those who did not across several continuous variables. The findings are summarized below the table.

**Table 3:** T-test Analysis Between No-Show and Show Groups. Following is the measure for each variable: Age (years), Weather (Fahrenheit), and Distance from clinic (miles). P-value threshold of < 0.05 was for statistical significance.

Two-Sample T-Test Results	Mean	Standard Deviation	t-Statistic	p-value
Age Comparison				
No-Show	40.73	14.08	-1.684	0.092
Show	42.85	12.48		
Weather Comparison				
No-Show	64.22	16.72	0.613	0.54
Show	65.03	16.1		
BMI				
No-Show	28.71	6.65	0.275	0.783
Show	28.85	6.61		
Distance from Clinic				
No-Show	39.98	50.92	-1.39	0.166
Show	34.49	31.85		

The mean age of no-shows (40.73 years) was slightly lower than that of shows (42.85 years). However, the difference was not statistically significant (t=−1.684, df ≈ 454, p=0.092). The standard deviations were 14.08 years for no-shows and 12.48 years for shows, indicating slightly greater variability among no-shows.

Weather conditions showed no significant difference between shows (mean: 65.03, SD: 16.10) and no-shows (mean: 64.22, SD: 16.72) (t=0.613, p=0.54).

The mean BMI for shows (28.85) and no-shows (28.71) was nearly identical, and the difference was not significant (t=0.275, p=0.783). The standard deviations were also similar, at 6.61 for shows and 6.65 for no-shows.

No-shows tended to live farther from the clinic (mean: 39.98 units, SD: 50.92) compared to shows (mean: 34.49 units, SD: 31.85). However, this difference was not statistically significant (t=−1.39, p=0.166).

Finally, Table 4 examines the associations between procedure categories and patient attendance (show vs. no-show). Of the 145 total appointments for breast procedures, 65 were no-shows, and 80 were shows (p=0.22). Subcategories of breast procedures showed no significant differences in attendance rates. For body contouring/abdominoplasty, a total of 138 appointments were recorded, with 75 no-shows and 63 shows (p=0.14). Attendance rates for subcategories were also not significantly different. However for facial procedures, significant differences were found in attendance rates (p<0.001). It revealed significant results, where facelift/blepharoplasty had six no-shows and 16 shows (p<0.001) and facial fracture follow-ups had 46 no-shows and 17 shows (p<0.001). No significance was seen for miscellaneous procedures like lesion removal, scar revision, and botox (p=0.48).

**Table 4:** Surgical Procedure Types Analysis Between No-Show and Show Groups. P-value threshold of < 0.05 was for statistical significance.

Procedure Category	No-Show	Show	Total	p-value
Breast Procedures	65	80	145	0.22
- Breast Augmentation / Mastopexy	46	46	92	1
- Breast Reduction	13	22	35	1
- Other Breast Reconstruction	3	3	6	1
Body Contouring / Abdominoplasty	75	63	138	0.14
- Abdominoplasty (Tummy Tuck)	31	31	62	1
- Panniculectomy / Excess Skin Removal	9	9	18	1
- Liposuction	13	7	20	0.13
Face Lift / Blepharoplasty	6	16	22	0
Facial Fracture Follow-Ups	46	17	63	0
Miscellaneous Procedures	33	30	63	0.48

Table 5 presents the results of the multivariate regression model. Male patients had significantly higher odds of missing their appointments compared to female patients (OR = 1.85, 95% CI: 1.12-3.06, p = 0.014). Post-operative patients were also more likely to miss appointments than new patients (OR = 1.92, 95% CI: 1.25-2.95, p = 0.003), while pre-operative patients showed an increased likelihood of no-show, though this did not reach statistical significance (OR = 1.45, p = 0.08). Weather conditions remained a significant factor after adjusting for other variables. Cloudy weather was associated with higher no-show rates compared to fair weather (OR = 1.67, 95% CI: 1.20-2.34, p = 0.002), whereas light rain did not significantly affect attendance (p = 0.38). Additionally, facial procedures were linked to significantly lower no-show rates compared to body contouring procedures (OR = 0.48, 95% CI: 0.29-0.79, p = 0.004). 

**Table 5:** Multivariate Regression Model for No-Show Rates. P-value threshold of < 0.05 was for statistical significance.

Variable	Odds Ratio (OR)	95% Confidence Interval (CI)	p-value
Male Gender	1.85	1.12 – 3.06	0.014
Patient Type			
Post-Operative	1.92	1.25 – 2.95	0.003
Weather			
Cloudy Weather	1.67	1.20 – 2.34	0.002
Procedure Type			
Facial Procedures	0.48	0.29 – 0.79	0.004

## Discussion

The results of this study provide insight into the various factors encountered by patients that contribute to a higher incidence of no-show appointments in a rural, plastic surgery clinic. Common reasons identified in the literature for missed appointments include forgetfulness, misunderstanding, or patients not receiving a reminder message [13,14]. The clinic staff attempts to contact patients a few days prior to the visit to remind them. There are several demographic and environmental factors that strongly influence no-show rates. These demographic and environmental factors are particularly pronounced in Appalachia, a region characterized by higher poverty rates, limited medical infrastructure, and challenging mountainous terrain. 

The current study revealed a statistically significant association between gender and no-show rates, with males being more likely to miss appointments than females (p = 0.0078). This finding aligns with existing literature to suggest that males, especially in rural areas, are more likely to miss their appointments [15]. Mander et al. found that male patients were 1.57 times more likely to miss a scheduled appointment compared to female patients. This finding may be explained by males typically having a more self-reliant attitude and are therefore more likely to be reluctant to seek medical care [16]. Women, in contrast, are more likely to cancel their appointments proactively [17]. These findings highlight the importance of addressing gender-specific challenges to reduce no-show rates, ultimately improving access to care.

Patients with self-pay insurance demonstrated significantly higher attendance rates compared to those with private insurances. This finding may reflect the financial commitment associated with self-pay services, which may serve as motivation for patients to prioritize attendance. Insurance status is often a predictor of missed appointments, where patients who have recently lost insurance coverage are more likely to miss [7,18]. Further, insurance type (whether private or public) and provider coverage are well-documented reasons why patients miss their appointments [2]. However, there is evidence in the literature to suggest that insurance status does not play a role in missed appointments. Dantas et al. analyzed factors associated with missed appointments in a bariatric surgery clinic and concluded that forms of payment were not a significant factor for no-shows, suggesting that other factors may play a more dominant role in attendance behavior [19]. Similarly, Chen et al. reported no significant difference in missed appointments between patients with private versus public insurance [20]. 

Adverse weather conditions were associated with increased no-show rates, particularly during cloudy or rainy days. This finding aligns with prior research regarding the impact of weather on patient attendance. In Appalachia, where long travel distances and mountainous terrain contribute to transportation challenges, weather conditions significantly disrupt patient attendance. There is a well-established positive association in the literature between inclement weather and missed appointments [21]. Remigio et al. reported an incidence ratio of 1.03 missed appointments per 10mm of rainfall and snow and a 29% higher risk of no-shows during wind advisories [21]. However, Treimstera et al. found no significance between weather and patient no-shows, suggesting that regions with milder weather patterns may experience a lesser impact on attendance rates [22]. Addressing inclement weather may involve flexible scheduling, telemedicine options, or rescheduling policies that accommodate weather-related barriers.

This study found no significant differences in age or BMI between the show and no-show groups. While these factors are often associated with healthcare behaviors in other populations, their limited impact in this study suggests that other variables, such as transportation and socioeconomic barriers, may be more significant barriers faced in Appalachia [9]. This finding contradicts current literature as odds of missing an appointment decreased with increasing age [14]. A study conducted by Shour et al. in a Wisconsin medical center found that appointments for patients aged 21-30 had the highest rate of no-shows (11.8%) [23]. Similarly, in a study by Mander et al., patients aged 45-54 were significantly less likely to attend [16]. There are fewer reported associations between BMI and no-show rates documented in the literature, however, BMI may play a role in patient attendance. Chapman et al. noted that fear of confrontation by healthcare providers regarding weight and a perceived judgment of lifestyle may lead patients to avoid attending medical visits [24]. Addressing patient weight can be a sensitive topic and some obese patients may delay or avoid healthcare due to the fear of weight being addressed [25]. The role of weight and BMI among plastic surgery clinics is an interesting topic and future studies warrant investigating these associations.

Contrary to expectations, distance from the clinic was not a significant predictor of no-shows. This finding may reflect the resilience of rural Appalachian patients who are accustomed to traveling long distances for healthcare. However, additional research is needed to explore whether travel-related cost, time commitments, or other factors influence attendance. A study conducted by Mielosyzk et al. reported that patients with increased driving distance to the clinic were more likely to no-show their appointment [7]. No-show rates were calculated to increase by 0.1% for every 5 miles of distance commuted [7]. There is varying evidence in the literature, however, regarding attendance and distance from the clinic, suggesting that there are potential differences in socioeconomic backgrounds among patients in rural settings compared to urban settings that influence attendance [15]. Interestingly, Menendez et al. reported that patients were more likely to miss their appointments if they lived in close proximity to their healthcare clinic [17]. This finding may be explained by a patient's perception of having an easier time rescheduling. Due to the varying evidence in the literature, further research is indicated to determine other socioeconomic factors that may contribute to patients travelling distances and their risk of appointment no-shows. 

Understanding the factors contributing to patient no-shows is crucial for guiding future clinical practice to alleviate these issues. Missed appointments are a significant healthcare burden leading to significant unwanted costs. Ultimately, missed appointments affect the patient’s health outcomes and impact the care of prospective patients by taking appointment slots. To address these factors, it is important to establish a strong provider-patient relationship to improve care accessibility [10]. It is essential to tailor appointment reminders and offer flexible scheduling for patients to improve patient attendance. Telemedicine may serve as a vital tool for patient attendance and the role of Telemedicine visits should be investigated within a plastic surgery clinic. Further, it may be advantageous to offer patients a financial incentive such as upfront payment for aesthetic services and bundling services together. 

This study provides an in-depth analysis of variables, including patient demographics, appointment details, and environmental conditions to offer better insight into the factors associated with patient no-shows. The limitations of this study include its retrospective design, which may impact its generalizability. Further, the small sample size is a limitation and future studies should include a larger sample size with additional Appalachian communities or other rural regions to provide greater insight into the barriers faced by patients in this region.

Future studies are warranted to examine the role of patient satisfaction, health literacy, and clinic-level interventions in reducing no-shows. Longitudinal research should assess the long-term impact of strategies such as telemedicine or financial incentives on appointment adherence. Future research should also analyze the optimal time period that patients should be scheduled out [23]. 

The results of this study demonstrate that no-show rates are influenced by a multifaceted interplay of demographic, and environmental factors. Among these, male gender, insurance type, and weather conditions emerged as significant predictors of appointment adherence, while variables such as age, BMI, and distance from the clinic showed mixed or non-significant associations.

## Conclusions

No-show rates are influenced by demographic and environmental factors. Higher rates of no-show appointments had a significant association with male gender, insurance type, and weather conditions, while variables such as age, BMI, and distance from the clinic showed mixed or non-significant associations. Future studies are warranted to determine the role of telemedicine on improving patient attendance within plastic surgery clinics.
